# Intraoperative Catastrophe during Benign Mediastinal Tumor Mass Excision: A Case Report

**DOI:** 10.7759/cureus.4941

**Published:** 2019-06-19

**Authors:** Akshay Kumar, Purandeo Persuad, Nimisha Shiwalkar

**Affiliations:** 1 Cardiothoracic Surgery, Lokmanya Tilak Municipal Medical College & General Hospital, Mumbai, IND; 2 Miscellaneous, The University of Kansas Medical Center, Kansas City, USA; 3 Anesthesiology, Lokmanya Tilak Municipal Medical College & General Hospital, Mumbai, IND

**Keywords:** mediastinum, germ cell tumor, mature teratoma, superior vena cava syndrome, extracorporeal circulatory support

## Abstract

Mature teratoma of the mediastinum poses a significant surgical challenge due to close vicinity to vital structures causing respiratory insufficiency or hemodynamic compromise. While the malignant variety of germ cell tumors (GCT) generally present with florid symptoms, benign teratomas are detected incidentally on imaging. Large teratomas presenting as mediastinal mass syndrome have additional difficulty in airway access. Herein, we report a case of a 40-year-old-female with no significant comorbidities presenting with rapidly progressing symptoms of chest pain, dyspnea, and superior vena cava (SVC) compression. Computed tomography (CT) scan of the neck and chest confirmed a large cystic lesion with marked compression of the great veins in the neck, arch of aorta, trachea, and proximal bronchial divisions. Airway access intraoperatively was done by awake fiberoptic bronchoscopy. However, sudden hypoxia and hemodynamic deterioration warranted emergency sternotomy. Adequate preoperative preparation, as well as standby extracorporeal circulatory support, led to successful and complete excision of the tumor. The patient had an uneventful recovery and extubated in the intensive care unit (ICU) the next day. Histopathology of the mass confirmed to be mature benign cystic teratoma. At six-month follow-up, the patient was completely asymptomatic without any complications. The impact of intraoperative catastrophe on the healthcare team can be immense. Inability to achieve a secure airway and the resultant hypoxia can result in permanent neurological damage. A multidisciplinary approach leading to adequate preoperative assessment, intraoperative preparedness for an emergency sternotomy with standby extracorporeal circulatory support due to the risk of mediastinal mass syndrome, were key features in the successful management of the patient.

## Introduction

Approximately 5% to 10% of all germ cell tumors (GCT) arise in non-gonadal sites, particularly in the mediastinum and retroperitoneum [1]; the most common primary mediastinal GCT being teratoma. Teratomas are categorized as mature, immature, and malignant. Benign teratomas are slow growing tumors, incidentally found on radiology, with only 50% showing symptoms at the time of presentation. Symptoms, if present, are generally due to compression of the mediastinal structure resulting in chest pain, cough, and dyspnea which is aggravated on lying supine [2-4]. Rare presentations include erosion into an adjacent bronchus, pericardium, and superior vena cava (SVC) syndrome [2,5]. Mature teratomas of mediastinum pose a significant surgical challenge due to its close vicinity to vital structures causing respiratory insufficiency or hemodynamic compromise.

## Case presentation

**Figure 1:**
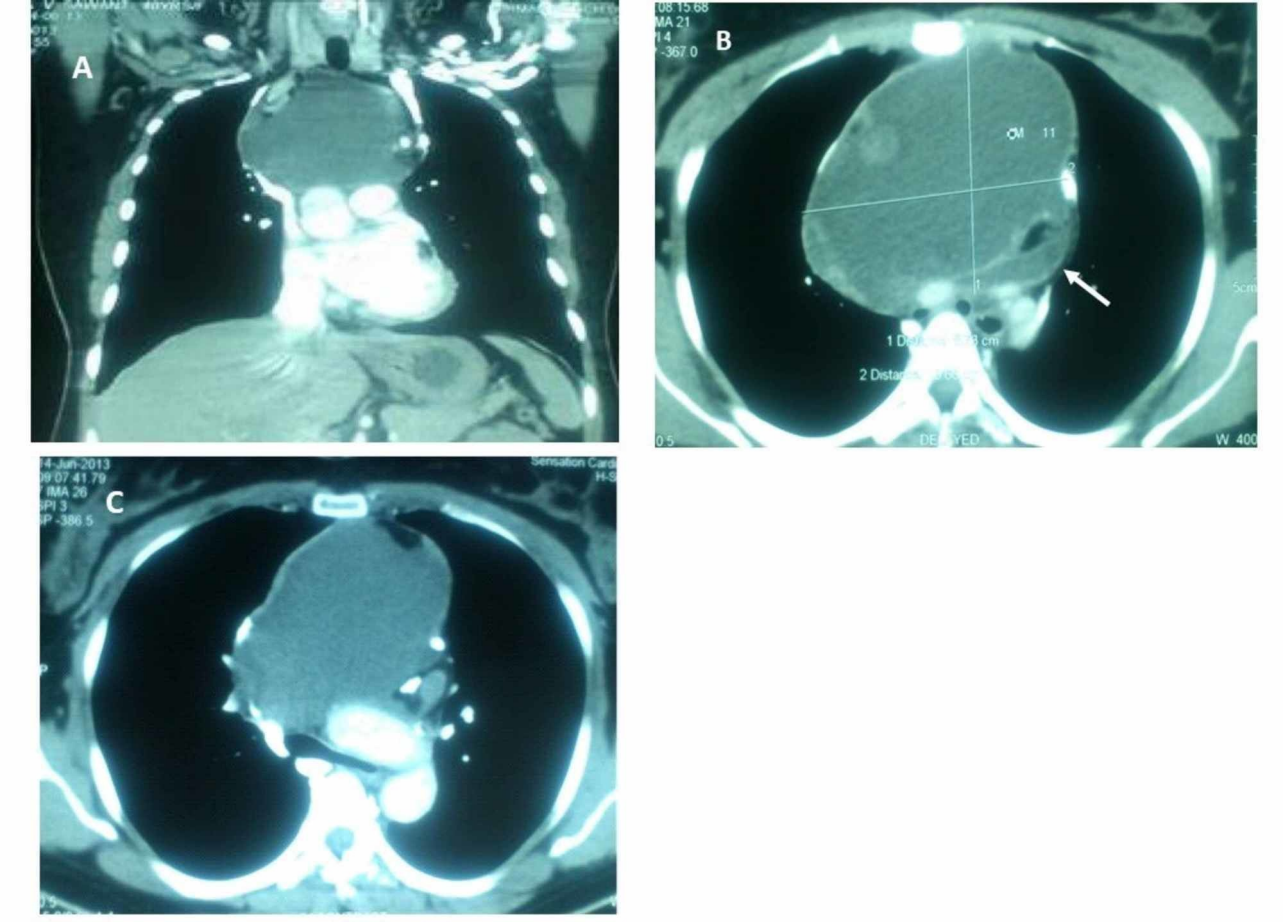
Computed tomography (CT) scan showing (A) tumor (white arrow) compressing the superior vena cava, and (B, C) trachea, right and left mainstream bronchus and arch of aorta

A 40-year-old female, previously healthy, presented to the outpatient clinic with intermittent cough without expectoration, breathlessness, difficulty in swallowing and facial puffiness for the last two to three months with gradual worsening over the past one month. The symptoms aggravated on lying in a supine position. Her past medical history was significant for hypertension and was on atenolol. On physical examination, her vitals were stable in the sitting position; it also revealed facial puffiness and distended neck veins. All her labs were within normal limit. Chest X-ray showed mediastinal widening. On contrast-enhanced computed tomography (CECT) of the chest, a large cystic lesion of size 14.4 x 10 x 8.6 cm was seen in the anterior mediastinum with marked compression of the brachiocephalic confluence, SVC, arch of aorta, proximal vessels of the neck and trachea, and proximal bronchial divisions (Figures 1A-1C).

**Figure 2:**
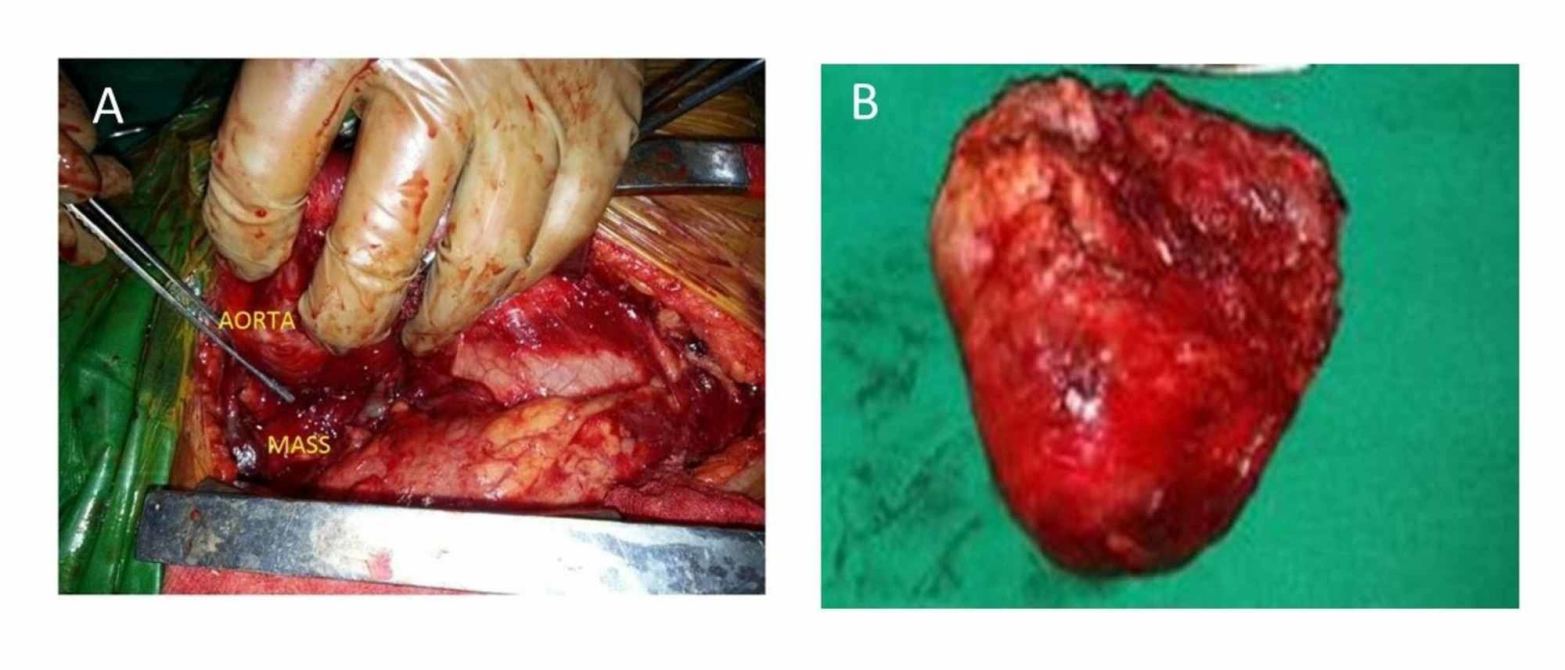
(A) Intraoperative picture of the tumor compressing the trachea; (B) specimen after complete excision

CT guided fine needle aspiration biopsy (FNAB) showed cheesy fluid aspirate with no atypical cells. A decision for tumor excision via a median sternotomy approach was planned. A thorough preoperative planning with regards to surgical access, use of intraoperative bronchoscopy, and patient positioning were done. Anticipating cardiorespiratory compromise as an intraoperative complication, preparation for extracorporeal circulation using femoral cannulation was kept ready. Large bore venous access was placed in the lower extremity, spontaneous ventilation was maintained, and fiberoptic bronchoscopy was performed to visualize the airway for any compression and facilitate intubation. However, after induction, the saturation suddenly dropped to low 50s. The cyst was aspirated from the left second intercostal space as an emergent measure, the neck was flexed, and head end elevated 30 degrees to facilitate ventilation. Emergency sternotomy was done and the tumor was totally resected thus relieving the compression (Figures 2A-2B).

**Figure 3:**
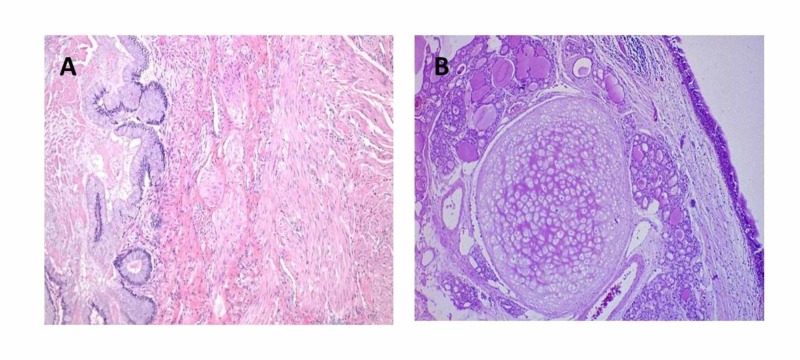
(A, B) Histopathology of the excised tumor showing mature epidermis, adnexal elements, bone and cartilage

Mediastinal and pleural chest tubes were placed for drainage. The patient was extubated in the operating room after the surgery and observed in the intensive care unit (ICU). The post-operative stay was uncomplicated. The mediastinal and left pleural drains were removed on post-operative day six and seven, respectively; the patient was discharged on day eight. Final histopathology report revealed a benign cystic teratoma, grossly showing multiple solid and cystic areas with bone cartilage and skin with hair follicle. Microscopic examination showed mature epidermis, adnexal elements, bone and cartilage (Figures 3A-3B). At six-month follow-up, the patient was completely asymptomatic and without any complications.

## Discussion

GCT is typically derived from ectoderm, endoderm, and mesoderm germ cells [1,3,6]. They can be classified into mature (cystic or solid, well differentiated, benign) or immature (malignant), gonadal or extragonadal in origin. Mature cystic teratomas are the most common mediastinal germ cell tumors representing 60-70% of all mediastinal GCT [2-3,7-8]. They are generally benign but have malignant potential [9]. Patients are usually asymptomatic and the tumor is found incidentally on chest X-ray [7,10]. CT scan is helpful in defining invasion of adjacent structures and thus assists in planning surgical intervention [7,11-14]. It can also detect fatty or cystic areas in mediastinal masses [13], but this information will not obviate the need for surgical resection to establish the final diagnosis.

As seen in Figure 1, a CT scan of the mediastinum in our patient established the presence of cystic mass with marked compression of nearby viscera. About 30%-59% of patients with mediastinal teratoma are asymptomatic at the time of diagnosis [12]. Symptoms most commonly observed include chest or back pain, dyspnea, and cough [6,8,12-13]. Spontaneous rupture of cystic mediastinal teratoma is rare but may result in serious complications [6,8,11]. Rupture reportedly occurs in up to 36% to 41% of mature teratoma cases [8,12].

Preoperative management includes bed rest, elevation of the head end and oxygen administration [15]. Intravenous lines in the neck should not be placed because jugular venous pressure may be markedly elevated and inadvertent hematoma formation may lead to airway compromise. For patients who require general anesthesia, continuous monitoring of gas exchange and hemodynamics during the induction process is critical along with maintenance of spontaneous ventilation and avoidance of muscle relaxant until the airway is secured and adequate gas exchange can be established with positive pressure ventilation. Anesthesia induction can be accomplished with either inhalation agents or intravenous titration of ketamine with or without other intravenous agents to maintain spontaneous ventilation. Awake fiberoptic intubation is also a recommended option but the rate of success will depend on the technique and experience of the anesthesiologist [16]. Normally, bleeding from these tiny vessels is self-limiting; however, in patients with SVC syndrome, venous pressure is elevated, and bleeding may be more pronounced.

Individuals with SVC syndrome may not be able to lie comfortably in a supine position for an extended period because of increased intracerebral venous pressure. As with all thoracic surgeries, proper positioning of the patient is of paramount importance. Tumors or cysts located in the anterior mediastinum are generally approached through median sternotomy approach via standard single-lumen endotracheal intubation. Double-lumen endotracheal tube for single-lung ventilation is reserved for thoracotomy incision and for all procedures performed using video-assisted thoracoscopic surgery (VATS). Additional exposure includes a hemiclamshell thoracotomy, which may be preferred for tumors in the anterior mediastinum with extensive involvement of the hemi-thorax [17-18]. A neck extension or supraclavicular extension can be incorporated with involvement that extends into the neck or subclavian vessels, respectively. A clamshell incision can also be used for tumors that extend into both hemi-thoraces.

Tumors that extend to adjacent structures necessitate resection of the thymus, pericardium, part of the lung, phrenic nerve, innominate vein as also the reconstruction of SVC. Post-operative care is like that for any routine thoracic surgery. Extubation can be performed after the procedure or in the post-anesthesia recovery area. Patients requiring prolonged ventilatory support should have pulmonary toilet to prevent atelectasis and clear any bronchial secretions. Patient-controlled analgesia (PCA) is one of the widely used methods of pain management. Thoracic epidural analgesia can provide good pain relief and reduce the need for postoperative analgesia [19].

Sternal wound complications although rare, are significantly elevated in emergency cases. Heilmann et al., in their multivariate analysis, revealed emergency sternotomy as an independent prognostic factor for all sternal wound complications (odds ratio (OR) 3.5, 95% confidence interval (CI), 2.0-6.3; p<0.001) [20]. When drainage from the chest tubes is less than 50-100 cc in a 24-hour period, no air leak is present, and the chest radiograph shows full pulmonary expansion with no collections on the operated side, the chest tubes may be removed. Patients who undergo resection of benign neoplasms can be followed up for three to six months postoperatively for wound healing and progression of patient activity. Complications are the same with any other surgery involving the mediastinum. Rarely, teratoma may rupture into tracheo-bronchial tree or result in SVC syndrome or pneumonia [8].

Prognosis after resection of a mediastinal tumor varies widely depending on the type of lesion resected. After resection of mediastinal cysts and benign tumors, prognosis generally is excellent [3].

## Conclusions

Complete surgical removal of a mediastinal teratoma is the treatment of choice, as it establishes the diagnosis, besides preventing life-threatening complications in many patients. As most mediastinal teratomas are benign, even a subtotal resection conserving adherent vital structures provides excellent long-term results. In the present era of modern surgical practices, excellent outcomes have become the rule. The interesting feature of our patient was an atypical presentation with rapidly developing symptoms despite the benign nature of the tumor. The anticipation of cardiorespiratory compromise during anesthesia induction, appropriate preparedness, and prompt responsiveness in case of any compromise as performed in this case, is helpful to prevent catastrophic events.
